# An Essay on the Natural, Chemical, and Medical History of Water, in Its Simple and Combined States; Including an Account of the Chemical Composition, and Medical Effects, of the Principal Mineral Waters in the United Kingdom of Great Britain and Ireland, and Also of the Continent of Europe

**Published:** 1825-12

**Authors:** Michael Ryan

**Affiliations:** Member of the Royal College of Surgeons, London, &c. &c. &c.; Physician at Kilkenny.


ART. II.—*An Essay on the Natural, Chemical, and Medical History of Water, in its simple and combined States; including an Account of the Chemical Composition, and Medical Effects, of the principal Mineral Waters in the United Kingdom of Great Britain and Ireland, and also of the Continent of Europe.* By MICHAEL RYAN, M.D. Member of the Royal College of Surgeons, London, &c. &c. &c.; Physician at Kilkenny.

WATER, when pure, is tasteless, colourless, and inodorous: it owes its fluidity to the presence of caloric; its natural state, like that of all other bodies, being solidity. It is composed of 85.7 of oxygen, and 14.3 hydrogen; or one volume of oxygen gas to two of hydrogen. Its weight is assumed as the standard of specific gravity: one cubic foot, at $60^\circ$ or $30^\circ$ of barometer, weighs 1000 ounces; or one cubic inch, 252.42 grains. Mr. Dalton is of opinion that water is a binary compound, of one atom of oxygen and one atom of hydrogen; and, adding the weights of these atoms together ($7.5 \times 1$,) an atom of water will weigh 8.5. Water is an essential constituent in the organisation of all living bodies, whether animal or vegetable; and it exists, in the solid form, in most of the mineral substances. When most pure, it is a compound, and, from its extensive solvent power, is scarcely to be found in a simple, pure, and uncombined state; for even in every fountain the water is impregnated with saline and earthy substances, which are generally insufficient to change the sensible qualities of the water, or to

I cannot understand this.

render it unfit for the ordinary purposes of life; and, if transparent, colourless, inodorous, tasteless, and not liable to spontaneous change, it is called pure. It remains liquid at the common temperature of our atmosphere, and assumes a solid form at 32°, expanding one-tenth with an immense force; and the gaseous at 212°, expanding 2000 times its ordinary bulk; or in vacuo at 90°; returning to its liquid state on resuming any degree of heat between these points. It can sustain the heat of 400° in Papin's digester. This fluid is capable of dissolving a greater number of natural bodies than any other in nature whatsoever; and, when saturated with one substance, it can take up a portion of a second, third, or fourth; during which solution a change of temperature takes place; and, when it enters into a state of combination with various solid bodies, it loses its fluidity, and forms hydrates or hydroxures. It is found throughout the earth, not only in its uncombined states of solid, fluid, and gas, but permanently united to a vast number of bodies, whether solid, fluid, or gaseous. During the solution of bodies, the bulk of the water changes, and its solvent power is increased by diminishing the pressure of the atmosphere. It is a most abundant ingredient in animal and vegetable substances. If we examine the embryo state of nature, we shall find much truth in this assertion: the hardest bones of animals were first an aqueous jelly, and the hardest wood was first a drop of viscid water. Watson, in his Chemical Essays, asserts that, if the hardest wood be converted into charcoal, it loses three-fourths of its weight, which is almost entirely pure water; and that great chemist, Dr. Kirwan, states "that grass loses, by drying into hay, two-thirds of its weight; or, if submitted to distillation, affords two-thirds of its weight of pure water."* Dr. Hales proved by experiment, "that a sunflower, weighing three pounds avoirdupois, and watered daily, perspired, or passed through it, twenty-two ounces each day; nearly half its weight of water. Thus Thales, the Milesian, is tolerably correct, when he denominated water the "omniseminaria," the seminary of all things.

This useful and necessary fluid presents itself to our notice in three distinct forms,—namely, in a fluid, gaseous or vaporous, and in a solid or frozen state. It descends in the forms of dew, rain, hail, or snow; or from the earth, which sends it forth in springs and rivulets. In the former case, the watery exhalations drawn from the sea and surface of the earth by the sun's heat, form clouds, whose particles, being afterwards condensed, fall in showers. In the latter, the water which falls on the tops of mountains, and other lofty situations, penetrates the earth, and, after passing downwards, breaks forth at some fissure or aper-

---

* Kirwan on Manures; an Essay read before the Royal Irish Academy.

ture, at a distance from its source. Water differs according to the source from whence it is derived, as rain, snow, river, ice, well, and fountain water; and also according to the strata through which it passes.

Snow and hail are said to be produced by the condensation of water, diffused through the different regions of the atmosphere, in different stages of aggregation : hail in the lower regions, by the condensation of large drops of water in the fluid state ; snow in the higher, by the crystallisation of very minute particles or of vapour. Hence it is that hail is not found about the polar regions, because water does not exist there in a fluid state. Snow may be produced by the admission of intensely cold air into a warm apartment. An instance is recorded of a ball-room in Russia, the window of which being opened, the humid vapour within was converted into snow, and fell, in this form, on all present.* Snow and hail are less transparent than ice, probably from a greater proportion of air mixing in their formation and composition, which, rendering their density less uniform, so far injures their transparency.

Water passes into the solid form at 32°, and then suffers a regular crystallisation, which is seen very evidently in the slow congelation of this fluid ; lines shooting from the sides of the vessel and from each other at a certain angle, that of 66° or 120°; and not less evidently in the radiated appearance than in a flake of snow. The figure of the crystals of ice, according to Hassenfratz, is that of an hexadral prism ; and of hail, according to M. d'Artic, of an octoedron. By the continuance of the crystallisation, the vacuities between the lines and the crystals are filled up, and a solid, brittle, and transparent mass is formed ; the state in which ice usually occurs. When caloric, or heat, is communicated to ice or snow at 32°, fusion takes place, accompanied with an absorption of caloric, from an enlargement of capacity. There is at the same time a diminution of volume ; water at 32° being denser than ice at the same temperature. The diminution of volume proceeds when the temperature is raised above 32°, and the greatest density of the water appears to be at about 40°. In receding from that temperature, either to a higher or lower, it expands in an increasing ratio as the temperature falls, the expansion continuing until it becomes solid ; and, in the very act of freezing, it exerts a very strong expansive power. The air which water holds dissolved, is expanded during freezing, and hence ice is porous; but the expansive force and enlargement of volume, as it approaches the freezing point, is owing to the new arrangement into which the particles, from their polarity, appear to

---

* STACK's Introduction to Chemistry Dublin, 1802.

pass. Water is suspended in the air in considerable quantity, which is said to be owing to solution; so that, were all that is dissolved in it precipitated, there would be probably enough to cover the earth's entire surface, not only to the height of thirty-two feet, but to an indefinite height. It has been limited to thirty-two feet in height, because the pressure of the air was intimately connected with the quantity of water contained in it; but daily observations show that this opinion is unfounded. There is a continued absorption of water into the air during a long summer's drought; and, on this hypothesis, the barometer should be continually rising; while, on the contrary, it is found stationary, during the whole time, at thirty inches or a little more; and, even when the drought is about to have an end, and the whole quantity of the water still contained, it becomes suddenly lighter, and the mercury will sink, perhaps, an inch: and, after the atmosphere has been discharging a fluid much heavier than itself for many days, instead of being lighter, it becomes heavier than it was before. From these remarks, and the large quantities of water proved to exist at the greatest depths below the surface, it is evident that the system of nature contained ample materials for effecting the universal deluge.

Mists and dews are generally greatest when the difference of heat in the day and night time is greatest, because in this case more water will be raised by the heat, and more falls by reason of the cold; so that both causes concur to increase the quantity of the dews. In cold weather, the breath of animals becomes visible, because there is not sufficient heat to dissolve the moisture which is exhaled from the lungs. This subject is most ably and fully illustrated in the Philosophical Essays of the Right Rev. Dr. Hamilton, late Lord Bishop of Ossory. Water exposed to the air in a shallow vessel disappears, being dissolved by the atmosphere. Saussure states the quantity of water in a cubic foot of air charged with moisture at 65° Fahrenheit, to be eleven grains. The quantity of water which may be extracted from one hundred cubical inches of air at 57° Fahrenheit, is 0.35 of a grain; but, according to Clemont and Desormes, at 54° Fahrenheit, only 0.236 of a grain can be detached by exposure to muriate of lime.

Instruments for measuring the degree of moisture of the air are called *hygrometers:* that invented by Mr. Wilson, of Dublin, has been much esteemed.*

Water enters into combination with various solid bodies, and entirely loses its fluid form, and such compounds were called *hydrates* or *hydroxures.* The pure alkalies, potass and soda, retain, for example, after fusion, one-fifth of their

* Thomson's Annals, ix. 313.

weight of water, which can only be separated by some body having a stronger affinity for the alkali. Water dissolves a great variety of solids, and during such solution a change of temperature takes place. Air is evolved during the solution of salts in water, which was partly contained mechanically in the salt, and partly in the water. The solvent power of this fluid is increased by diminishing the pressure of the atmosphere. At a temperature below 32°, ice passes into vapour when exposed to the atmosphere, perhaps from the affinity exerted towards it by the gases which compose atmospheric air. Water also evaporates slowly at a low temperature; the evaporation becomes more copious as the temperature is raised; it boils at 212° under the mean atmospheric pressure, or that which is equal to the pressure of 29½ inches of mercury, and in vacuo at 90°. In passing at any temperature into vapour, it absorbs a quantity of caloric, which becomes latent, and is less as the temperature of the water increases. When vapour of water, as steam, is fully formed, it is transparent and invisible; but, when condensing, a degree of opacity arises, from the approximation of its particles. At the temperature of 212°, it occupies 2000 times the space which it did in the form of water, and appears to have a specific gravity, to that of the atmospheric air at the same temperature, as 10 to 14. The elastic force which it exerts is increased by every elevation of temperature, and, according to Mr. Dalton's experiments, nearly in geometrical progression. The elasticity of steam thus increasing at a higher ratio by elevation of temperature, is a mechanical agent of great power, and is productive of immense force, under the most complete control and adaptation, in the steam-engine, one of the most perfect and valuable instruments of human invention.

### *On the Medical Effects and Uses of Water.*

The human body is composed of solid and fluid parts, a structure which exists in all animated nature. Water was the first and most natural fluid which was employed by man and animals to afford such fluid particles, and, forming so large a portion of ingesta, must have a powerful and constant agency on the human body. Thus, when the heat of the body is increased, an aqueous fluid is copiously exhaled through the skin; and, on the evolution of caloric necessarily attendant on its transition from a gaseous to a fluid state, the reduction of temperature principally depends. Insensible perspiration is, perhaps, little else than pure water with a very minute quantity of salt; and the obvious uses of this copious excretion seem to be, not only to remove a superabundance of water from the system, but especially, by carrying it off in a gaseous form, to constitute the great cooling process, and thus keep in proper check the production of heat by the lungs. As an aliment, its use is to hold in solution, and convey in a proper form, the various substances which constitute the solid food of animals. This fluid itself is a very necessary aliment, and is the chief mean of preserving the due proportion of fluid to solid matter, on which the preservation of life and due performance of all the functions depends. It will have different effects on the stomach according to the different temperatures: thus, at 60 it has little effect; between 45° and 60° produces cold, improves the stomach, and is tonic; below 45°, it produces a greater degree of cold in that organ and all over the body, is astringent and sedative; and between 60° and 80°, it causes nausea, relaxing the fibres of the stomach. Water taken into the intestines dilutes the chyle, and soon acquires the temperature of that fluid. If taken in great quantity, it increases the circulating fluids, induces plethora, apoplexy, dropsy, and a phlogistic diathesis. Its effects producing plethora and fluidity, are very transitory. It dilutes the contents of the stomach and intestines, often diminishing their acrimony, It acts on the kidneys and skin, and the quantity of secretion will be equal to the quantity taken into the body, though the equilibrium is sometimes destroyed, for the cutaneous exhalation may exceed that of the kidneys, and *vice versâ.*

As a diluent in internal hemorrhages, its temperature should not exceed 45°, while in fevers it may be 60°; except in the cold stage, when the thirst should be allayed by warm water, or some other bland fluids. In the latter complaints, it quenches thirst, reduces heat, and promotes perspiration; and the feelings of the patient afford a good guide whether it should be taken hot or cold. In cholera morbus and dyspepsia, where acrid matter exists in the primæ viæ, water is naturally desired. If taken when the body is overheated, it may cause inflammation of the stomach, which will speedily be followed by gangrene or death; and this by the abstraction of caloric,—an effect which will be prevented by keeping up, or increasing, the temperature of the body after the application of the cold.

A question has been discussed by learned men, whether water was pure, or could be changed into different other substances, as earth. The Hon. Mr. Boyle, and Margraff, the eminent chemist of Berlin, asserted that water was converted into earth by distillation; but Boerhaave is said to have distilled water 500 times, without any sensible alteration; while the unfortunate Lavoisier weighed the glass vessel before this process, and found that it lost in weight as much as the earth produced,—which fact put an end to the controversy. Yet it is now admitted that every kind, even of spring water, is impregnated with saline and earthy substances, which causes its hardness, which is said to depend on sulphate of lime. The other ingredients are carbonate of lime, muriate of lime, and muriate of soda; and the water is purified from them by distillation. Pure water has been procured by a simple apparatus, adapted to a ship's boiler, from sea water; whereby one of the most distressing wants is often supplied.

Rain water is said to be next in purity to distilled, from which it scarcely differs as to specific gravity; is most pure when collected in the open fields; it is that which has undergone a natural distillation from the earth, and is condensed in the form of rain: it is so pure as to be equal to distilled water for every purpose, except for the nicer chemical experiments. The foreign contents of rain water will vary according to the state of the air through which it passes, and, though they often cannot be detected by chemical examination, they frequently separate by spontaneous decomposition or long standing: hence rain water, if long kept, especially in warm climates, acquires a strong smell, becomes filled with animalculæ, and soon approaches to putrescency. The impregnations of rain water are said to be nitric and muriatic acids, muriates of soda and lime, atmospheric air 3½ inches to every 100 cubic inches of water, rather more oxygenous than common air, some carbonic acid gas; and, in large towns, a quantity of calcareous matter, especially sulphate of lime, which is derived from the mortar of the houses; also soot and other impurities.

If deprived of air for any time, water becomes putrid in some measure, and may be purified by agitation in the air, which gives it a brisk and pleasant taste, or by boiling, which separates the noxious air, or by filtration. Muriate of lime may be precipitated by barytic water; it is to be exposed to the air, until the precipitate is totally deposited; after which it may be regarded as pure, and fit for pharmaceutical purposes.* Alum also purifies muddy water; one pound of which is put into each cask of wine in Paris, and is also used by publicans, to purify spirituous liquors.

Ice and snow waters are said to be equal in purity to the last described. When first melted, they do not contain air, but only after standing for some time. In cold climates, or in the highest latitudes, thawed snow forms the drink of the inhabitants during the winter; and the vast masses of ice which float on the polar seas afford an abundant supply to the mariner. Captain Cook kept his sailors in good health, with ice water as their drink. Snow water has long lain under the imputation of causing scrofulous swellings in the neck, which so deform the

---

* Morveau An. xxiv. 320.

inhabitants of the Alpine valleys: but this opinion is not supported by authenticated facts; for this disease is frequent in Sumatra, where ice or snow are never seen, and it being quite unknown in Chili and Thibet, though the rivers of those countries are supplied with water from the snow on the mountains. Fish cannot live in snow water; neither does it boil vegetables well.

Fountain water is that which exudes from beneath the soil, and which must be various, as to its foreign ingredients, according to the strata through which it passes. It is distinguished as hard and soft, sweet or brackish, clear or turbid. As this water has its source at some depth from the surface, and thus so much removed from the action of the atmosphere, its temperature is warm during every vicissitude of season, and always higher than the freezing point. Some springs are hot, exceeding the summer heat; and such warmth is independent of the temperature of the atmosphere, and varies little either winter or summer. Every spring water is hard, which is owing to saline impregnations, which do not impair the taste of the waters: these are generally in the proportion of five grains to each pint, which renders a water unfit for washing with soap. To free a hard water from its ingredients, boil it, and allow it to cool, and then drop into it an alkaline carbonate; lastly, filter it. Such water, however, cannot be used for pharmaceutical purposes. Others add potass to hard water, which unites with the acid of the earth, which is separated. Spring water often incrusts the inside of any tube through which it has to pass; and, as this water never becomes putrid under any circumstances, it was preferred by the ancients. Well and pump water are nearly similar.

River water is softer, and more free from earthy impregnations, than spring, and contains less air of any kind; for, by the agitation of a long or rapid current, and an increase of temperature, it is deprived of common air, and also of carbonic acid, with the lime which it holds in solution. The specific gravity thereby becomes less, and the taste not so harsh, but less agreeable; and out of a hard spring a stream of sufficient purity may issue, which is fit for most purposes where soft water is required. The water in large rivers, as the Thames or Liffey, is not so impure as is generally supposed; for it appears, from chemical analysis, that the impurities communicated by manufactories to these waters have little, if any, effect in changing the qualities of them. However, as many impurities of a grosser kind are wilfully or inadvertently thrown into them, cisterns have been wisely established for filtration, and pumps for affording a pure water.

Stagnant waters contain the greatest impurities, as they are generally filled with the remains of animal and vegetable substances undergoing decomposition, during which they partly become soluble in water; and thus affording a rich aliment to the succession of living insects and plants, which are constantly supplying the place of those that perish. From want of agitation in these pools, vegetation proceeds without interruption, and the substances become covered with conferva and other aquatic plants. The taste of such waters is vapid and disagreeable, and destitute of coolness, and they are justly condemned as unfit for use in human food.

A few of the opinions of the early physicians on the preference in the use of these waters, may be mentioned. Hippocrates,* Galen,† and Avicenna,‡ "recommended fountain water in preference to all others;" while Celsus§ thought "that rain water was lightest, then fountain, next river, then pit-well, then snow and ice waters, next lake, and most inferior was marsh water." Ætius said, "rain water was lighter than all, and most easily changed;" and Hippocrates praised river water, "because it was light, sweet, and limpid." Vitruvius‖ asserted, "that water collected from showers possessed more salubrious properties than that of the lightest fountains; for it was purified by the air, and descended thus to the earth." Averroes¶ preferred "rain to fountain water, as vegetation was improved by showers;" and Aristotle maintained, "that sea-fish suffered much when there were not showers." Mankind, in general, prefer fountain or spring water, as it never undergoes any change.

When the ingredients are such as to change the sensible and physical qualities of water, it is called *mineral*. It requires an intimate knowledge of the action of bodies on each other, and the utmost nicety in the manipulations, to analyse them; the quantity of water on which the chemist has to operate being so minute. The substances found in mineral waters may be divided into the gaseous, the acids, the alkalies, the earths, the compound salts, and the metals.

1. Atmospheric air is contained in almost all mineral waters, in the proportion of one twenty-eighth of the volume of the water; and was first discovered in them by the Hon. Mr. Boyle.

2. Oxygen gas was first discovered in these fluids by Scheele; it is not contained in the same fluid with sulphuretted hydrogen, or the compounds of iron.

3. Nitrogen gas was detected in the waters of Bath by Dr. Priestley; in those of Buxton, by Dr. Pearson; of Harrowgate, by Dr. Garnet; and of Lemington, by Dr. Lambe. If not found in every sulphureous water, it is in all those containing a hydro-sulphuretted alkali.**

---

* A.C. 460. † 131. ‡ A.D. 980. § Lib. vii. c. 2.

‖ A.D. 350. ¶ A.D. 1150.

** M. Anglada, *An. de Chim.* xviii. 113.

4. Carbonic acid gas is a very common ingredient in them, and was first discovered by Dr. Brownrigg: its quantity varies considerably. Dr. Higgins states, " that 100 cubic inches of Pyrmont water contain 160, and according to Westrumb 187, inches of this gas."

5. Sulphureous acid is said to have been found by Vandellius, near the town of Latera, thirty miles from Viterbo in Italy.*

6. Sulphuretted hydrogen is very common in mineral waters, and some say was first discovered by Scheele; others, as Fourcroy, say by Rouelle and Bergman.

7. Boracic acid has been found in some of the waters of Italy.

8. Nitrous and muriatic acids have been found in a pure or uncombined state.

9. Soda is the only alkali said to exist in a free state in those waters; it was detected by Dr. Black, in the waters of Geyser and Rykum, in Iceland.

10. Lime, in its pure state, is said to be found in some of these waters.

11. Silicia, said to be found in waters of Geyser, Rykum, Carlsbad, and Pongues. It was said to be held in solution by soda, which is not the case; for the alkali in such waters is too trivial.

Sulphuric, nitric, muriatic, and carbonic acids, and sulphuretted hydrogen, are frequently combined with potass, soda, lime, magnesia, and oxyde of iron; and more rarely with barytes, alumina, and oxyde of copper. Subborate of soda was detected in the lakes of Thibet; but such waters are unfit for medical use.

12. Sulphate of soda often exists in mineral waters; was described by Bodalue, in the Memoirs of the Royal Academy of Paris, 1724, as existing in large quantity, and like icicles, near Madrid. Dr. Nichola Andrea describes a spring in Calabria, in which it exists so copiously, that he would recommend the manufacturing of it.

13. Sulphate of lime is a common ingredient.

14. Sulphate of magnesia is very commonly found; said to be in the proportion of ten pounds to eighty gallons of the Epsom waters, by Rutty.

15. Sulphate of iron was found in the waters in Denmark, by Bergman, and of Hasley Green, by Dr. Garnet; also in volcanic springs.

16. Sulphate of copper is only found in waters which issue from copper mines; and the learned Dr. Rutty observes, that all such waters are emetic and purgative, and generally unfit for use.

17. Sulphate of alum, found in Somersham waters by Dr. Layard, and in Ballycastle by Dr. Rutty.

18. Nitrate of potass has been detected in the waters of Hungary; it is rare.

19. Nitrate of lime, discovered by Dr. Home; said to exist in the waters in the desert of Arabia.

20. Nitrate of magnesia is rarely found in any water.

21. Muriate of potass, said to exist in the waters of Sweden.

22. Muriate of soda is a most common ingredient in mineral waters.

---

* De Thermis *Agri Patavini*, 1761.

23. Muriate of ammonia has been found in the lakes of Italy and Siberia.

24. Muriates of lime and magnesia are frequently found.

25. Muriate of barytes and alumina, rare; seen by Bergman and Withering.

26. Muriate of manganese, found by Dr. Lambe, in the water of Lemington, and by Dr. Scudamore in that of Tunbridge; also by Scheele: properties unknown.

27. Carbonate of potass, detected in the waters of Berlin, by Margraff.

28. Carbonate of soda, found in waters of Hungary, Tripoli, and Egypt; very common.

29. Carbonate of ammonia has not been found hitherto; if at all, it is very rarely.

30. Carbonate of lime, with an excess of carbonic acid; the most common ingredient in mineral waters.

31. Carbonate of magnesia is also often held by excess of carbonic acid.

32. Carbonate of alumina discovered by Westrumb; very doubtful.

33. Carbonate of iron is very commonly found, and mostly held in solution by carbonic acid.

34. Hydrosulphuret of soda has been sometimes observed; hydrosulphurets are not uncommon, in sulphureous waters.

35. Hydrosulphuret of lime is rarely found. The burning-wells in Brosley and Wigden, in Lancashire, contain a fine bituminous vapour, that arises and readily takes fire; was described by Shirley, in the Philosophical Transactions, 1759.

From the great variety of ingredients which thus exist in mineral waters, and the various effects they have on the human body, it appears that nature has done much for us, in supplying mankind with many safe and efficacious remedies, prepared to our hands, in a more elegant manner than modern chemistry or pharmacy can rival. The following is a list of some of the most celebrated mineral waters in Europe, alphabetically arranged.

Abcourt, St. Germain's, France, contains carbonate of iron and soda; is diuretic and aperient, and used in dropsy, jaundice, visceral obstructions, and eruptions.

Aberbrothick, Forfar, Scotland; a carbonated chalybeate, used in dyspepsia.

Acton, Middlesex, contains sulphate of magnesia and muriate of soda.

Aghaloo, Tyrone, contains sulphur, carbonate of soda, and aperient salts; used in scrofula.

Aix-la-Chapelle, Germany, contains carbonate of lime, muriate and carbonate of soda, and sulphur; is diaphoretic and purgative, and has been used in heartburn, asthma, ague, excessive menstruation; externally, in rheumatism, palsy, tremors, contractions, tumors, and cutaneous diseases.

Aix, in Provence; used in piles, diseases of the kidney and bladder, in fluor albus, excessive menstruation, and gonorrhœa; is somewhat similar to Blombieres.

Albermarle, or Aumale, Rouen, contains carbonate of iron.

Alford, or Awford, Somersetshire, is saline and strongly purgative.

Annfield, Burrisoleigh, Tipperary, an excellent simple chalybeate.

Alvenau, Switzerland, is famous for bathing.

Annaduff, Leitrim, is a sulphurous water.

Antrim, Ireland, contains muriate of soda, carbonate of lime, and bittern; has been used in dropsy, scurvy, jaundice, and visceral obstructions.

Ashwood, Fermanagh, is a saline and sulphurous water.

Ashton, Wiltshire, is a carbonated chalybeate water.

Athlone, Roscommon, is a simple, weak chalybeate.

Askeron, Yorkshire, is a saline and sulphurous water.

Athimonus, Leitrim, strongly sulphurous; much used in scrofula.

Austria is said to contain mineral waters, at Sellrain, Merain, Sexton, Prax, Agums, Brutz, Rabi, Pei, Stiria, Carinthia, and Carniola.

Baden, Germany, is a hot sulphurous water, resembling Aix-la-Chapelle.

Bagnigge, Middlesex, contains sulphate and muriate of magnesia; is aperient.

Baia, Italy, is a strong sulphurous water.

Balemore, Worcestershire, contains carbonate of iron; is strongly tonic.

Balaruc, near Montpellier, contains purging salts; is aperient and diuretic; has been employed in jaundice, palsy, scrofula, in diseases of the kidney and bladder; and externally in cutaneous affections.

Ballycastle, Antrim, is a chalybeate and sulphurous water; its effects are like Balemore.

Ballynahinch, Down, contains iron, carbonic acid, and sulphur; has been praised in scorbutic and dyspeptic cases. There is also a pure chalybeate in its vicinity.

Ballyspellan, Johnstown, Kilkenny, contains iron, carbonic acid, and muriate of soda; has been greatly esteemed in dyspepsia, chlorosis, lowness of spirits, and visceral obstructions.

Bagniers, Upper Pyrenees, said to be similar to Aix-la-Chapelle.

Bagnols, Guard, France, is a tepid, sulphurous water, and used in phthisis, affections of kidney, psora, palsy, and rickets.

Bandola, Italy, contains carbonic acid, iron, soda, and sulphur; is aperient, diaphoretic, and diuretic.

Bandon, Cork, is a simple chalybeate water.

Baregès, France, contains muriate and carbonate of soda, lime, and sulphur; its temperature 115°; is said to be similar to Bath. It has been used in nervous, œdematous, and cutaneous affections; in visceral obstructions, in rheumatism, chlorosis, and consumption; is aperient and diuretic.

Barnet, Hertfordshire, contains sulphate of magnesia and carbonate of lime.

Barrowdale, Cumberland, contains muriate of soda in great quantity, and carbonate of lime.

Bath, Somersetshire, contains carbonic acid, azotic gas, sulphate and muriate of soda, siliceous earth, selenite, carbonate of lime, and oxyde of iron; temperature, 112° to 116°.* It is diuretic, sudorific, and aperient; but, if drank in small quantities at the commencement, it induces constipation and stupor. It has been employed in diseases of the stomach, liver, bowels; in gout, rheumatism, and partial paralysis; in chlorosis, and, according to the excellent Treatise of Dr. Barlow,† in chronic eruptions.

Bologne, near Calais, contains carbonate of iron.

Bigova, Russia, a similar water to the last named.

Bonnes, Lower Pyrenees, similar to Bareges; temperature, 102°.

Bourbon l'Archambaud, near Moulins, is saline, and has been used in diseases of liver, jaundice, bowel affections, and apoplexy.

Bourbonne Lancy, is aperient, diuretic, and emmenagogue; has been used in cachexia, diarrhœa, fluor albus, œdema, and asthma. Externally, in palsy, tremors, rheumatism, and cutaneous affections.

Bourbonne, France, is a warm, saline, and sulphurous water.

Borset, near Aix-la-Chapelle, a warm, alkaline, and sulphurous water; 132°.

Brentwood, Essex, contains sulphate of magnesia and lime; purgative.

Bristol, Somersetshire, contains lime, muriate of soda, sulphate of magnesia, and selenite; temperature, 74°. It has been considered as a specific in consumption; allays hectic; is used in bilious diarrhœa, dysentery, diabetes, calculous and cancerous disorders, and fluor albus.

Bromley, Kent, a carbonated chalybeate; is diuretic and corroborative.

Broughton, Yorkshire, said to be similar to Harrowgate.

Brownstown, Kilkenny, contains carbonate of iron, lime, magnesia, and muriate of soda. It has been used with advantage in stomach, liver, or other visceral diseases; in calculous and uterine disorders; and against tænia lumbricus.

Buxton, Derbyshire, contains muriate and carbonate of soda, and lime; temperature, 82°; is praised in gout and rheumatism, and in diseases where bathing is useful.

Buda, in Hungary, is famed for its excellent baths.

Buzot, Spain, is a warm chalybeate water.

Caldas, Portugal, contains sulphur, gas, argilla, and muriate of soda; it is famed for chronic rheumatism and dyspepsia.

Calabria, Italy, a saline water, containing sulphate of soda.

Cape Clear, a saline, purgative water.

Carlsbad, Germany, a warm, alkaline, aperient water.

Caroline Baths, Bohemia, contain iron, carbonic acid, carbonate of lime, muriate and carbonate of soda, carbonate and sulphate of magnesia; are used in scrofula, dyspepsia, &c. in baths.

---

* Saunders on Mineral Waters. † Barlow on Bath Waters, 1822.

Carrickfergus, Antrim, from its blue colour, said to contain copper.

Carrickmore, Cavan, contains carbonate of soda; is aperient.

Cartmel, Lancashire, contains muriate of soda and Epsom salts.

Castlecomer, Kilkenny, situated in the beautiful and classic demesne of the Dowager Countess of Ormond and Ossory. It was analysed by the late Professor Higgins, of the Royal Dublin Society, and was found to contain iron, carbonic acid, and muriate of soda; results which I observed from my own experiments: and Drs. Wade, Garnet, and Ryan, thought it one of the best chalybeates in Ireland.

Castleconnel, Limerick, contains carbonate of iron; is said to resemble the German Spa, and is very much frequented for many years.

Castle[illegible]d, Rosshire, Scotland, contains carbonate of soda and sulphur.

Castlemain, Kerry, contains iron, sulphur, and carbonic acid.

Cawley, Derbyshire, contains sulphate of magnesia and sulphate of lime.

Cawthorp, Lincolnshire, contains iron, carbonic acid, and carbonate of soda; is aperient and antacid.

Cavan Spa, is a chalybeate.

Cauteries, Upper Pyrenees, near Bagnieres, is a warm water; temperature, 102° to 120°. Is much praised in acidity of the stomach, asthma, consumption, suppression of menses, and cutaneous affections.

Chadlington, Oxfordshire, contains carbonate and muriate of soda, and sulphur; is an aperient.

Chaude Fontaine, Germany, same as Aix-la-Chapelle.

Cheltenham, Gloucestershire, contains calcareous earth, iron, sulphate of magnesia, and muriate of soda; is aperient and tonic, and much used in dyspepsia, bilious complaints, scurvy, and gravel.

Chippenham, Wiltshire, contains carbonate of iron.

Clashmore, Waterford, said to contain sulphate of iron, by Elliot.

Cleves, Germany, similar to Pyrmont.

Clifton, Oxfordshire, near Bristol; to which it is similar.

Clonmell, Tipperary, a sulphurous water, much praised for scrofula.

Cobham, Surrey, contains iron and purging salt.

Codsalwood, Staffordshire, resembles Askeron water.

Colchester, Essex, contains sulphate of magnesia and carbonate of lime.

Coalcullen, Kilkenny, is a carbonated chalybeate.

Cork county contains many mineral waters, not given in this account, of minor note. (See my "Treatise on the Mineral Waters of Ireland," 1824, Longman.)

Corstorphine, Mid Lothian, contains sulphur and sulphate of magnesia.

Corville, Roscrea, Tipperary, a simple chalybeate water.

Coventry, Warwickshire, contains carbonate of iron and purging salt.

Cransac, Aveiron, contains carbonate of iron and sulphur.

Crosstown, Waterford, contains sulphate of iron; is diuretic and purgative.

Daswild Bad, Germany, contains carbonate of iron and saline matter; in visceral diseases.

Dax, near Bayonne, similar to Aix-la-Chapelle; discharges 543 cubic feet of water in fifteen minutes: is exhibited in affections of the kidney, in asthma, palsy, and rheumatism, in baths.

Deddington, Oxford, contains iron, sulphur, carbonated earth, muriate and carbonate of soda; greatly praised in cutaneous diseases.

Derby, Derbyshire, contains carbonate of iron.

Derryinch, Fermanagh, contains sulphur and carbonate of soda.

Derrindaff, Cavan, contains sulphur and purging salt.

Derrylester, Cavan, a strongly sulphurous water.

Digne, Lower Alps, warm, saline, and sulphurous; aperient and diuretic. Is used in dyspepsia, scrofula, asthma, visceral obstructions, and eruptions.

Dog and Duck, near London, contains sulphate of magnesia and muriate of soda; is said to be a cooling purgative.

Doneraile, Cork, a chalybeate.

Drigwell, Cumberland, similar to Deddington.

Driburgin, Westphalia, a saline chalybeate.

Dronisnamullock, Leitrim, a very strong sulphurous water.

Droppingwell, Yorkshire, contains carbonated earth; is astringent and tonic.

Drumasnane, Leitrim, one of the strongest sulphurous waters in Ireland.

Dudley, Worcestershire, sulphureous and chalybeate.

Dublin salt-springs, Francis-street, and Hanover-lane waters.

Dulwich, Kent, contains muriate of soda and sulphate of magnesia.

Dunnard, Dublin, a simple carbonated chalybeate.

Dunse, Scotland, contains iron, muriate of soda, and bittern.

Durham, contains sulphur and muriate of soda. A saline spring there.

Egra, Bohemia, said to be similar to Cheltenham.

Ems, Germany, famed for sulphurous baths.

Emsem, Germany, properties not described.

Enghiln, Netherlands, contains sulphuretted hydrogen.

Epsom, Surrey, contains muriate and sulphate of magnesia.

Fairburn, Rosshire, Scotland, contains sulphur and sulphate of soda.

Fahara, Switzerland, famed for baths.

Felstead, Essex, similar to Islington.

Filah, Yorkshire, is powerfully diuretic and aperient.

Forgès, Rouen, contains carbonate of iron.

Frankfort, Germany, is similar to Harrogate.

Gainsborough, Lincolnshire, contains sulphur, iron, and Epsom salts.

Geyser, Iceland, contains pure soda.

Galway, is similar to Tunbridge.

Garryhill, Carlow, a chalybeate.

Glastonbury, Somersetshire, similar to Clifton.

Glendy, Kincardine, Scotland, similar to Peterhead.

Granshaw, Ireland, similar to the German Spa.

Gran, Hungary, not described.

Grossal, Germany, a calcareous water.

Haigh, Lancashire, contains carbonate and sulphate of iron; is emetic.

Hampstead, Middlesex, similar to last named.

Haubridge, Lancashire, similar to Scarborough; is less aperient

Hanleys, Shropshire, contains purging salts, is aperient.

Harrogate, Yorkshire, discovered 1571, by Captain Slingsby; found to contain sulphur, muriate of soda, and purging salt. It is alterative and purgative; destroys worms; is praised in scurvy, scrofula, palsy, and chiefly in cutaneous diseases.

Hartfell, Scotland, contains sulphate of iron; is astringent and tonic, and used in all internal hemorrhages.

Hartlepool, Durham, contains sulphur, iron, and carbonic acid.

Healinglake, Cavan, cures scrofula, and is attested by many respectable persons.

Holt, Wales, is mildly purgative, and used in ulcers and eruptions.

Jerpoint Abbey Spa, Kilkenny, is a strong sulphurous water.

Italy contains many sulphurous and warm springs, of little note.

Joseph's-well, Surrey, contains a large portion of sulphate of magnesia.

Johnstown: see Ballyspillan.

John's-well, Kilkenny, same as Johnstown; not so strong.

Ilmington, Warwickshire, contains carbonate of iron and soda.

Inglewhite, Lancashire, a sulphurous chalybeate.

Islington, near London, contains carbonate of iron; is tonic and diuretic.

Kantusk, Cork, contains iron and sulphur; used in scrofula and eruptions.

Kedlestone, Derbyshire, is similar to Harrogate.

Kensington, near London, is similar to Acton.

Kilbrew, Meath, similar to Shadwell.

Kilburn, England, a sulphurous and saline water.

Kilcoran, Clare, a chalybeate.

Kilagee, Down, a chalybeate.

Killashen, Fermanagh, a sulphurous water.

Kileshan, Queen's County, a weak chalybeate.

Kilkenny College and Canal Spas, sulphurous and chalybeate.

Kilroot, Antrim, is similar to Barrowdale.

Kinalton, Nottinghamshire, a saline aperient water.

Kilmainham, Dublin, a sulphurous chalybeate.

Kincardine, Scotland, similar to Peterhead.

Kingscliff, Northamptonshire, similar to Cheltenham.

Kirby, Westmoreland, a saline chalybeate.

Knaresborough: see Droppingwell.

Klintschyselo, Russia, warm springs at.*

Knowsley, Lancashire, same as Scarborough.

Kuka, Bohemia, contains carbonate of soda; is diaphoretic and sialagogue.

Lancaster, similar to Tunbridge.

Langeac, Upper Loire, a cold acidulous water.

Latham, Lancashire, similar to Tunbridge.

Leuck, Switzerland, not described chemically.

---

* Clarke's Russia.

Llandrindad, Wales, a sulphurous chalybeate.

Lucca, Italy, a warm spring.

Llangybi, Wales, properties unknown.

Lisbeak, Fermanagh, strongly sulphurous.

Lemington, Warwickshire, contains muriate of soda and carbonate of lime; is strongly aperient, and much frequented.

Leez, Essex, same as Islington.

Listerlin, Kilkenny, a chalybeate.

Loansbury, Yorkshire, is sulphurous and aperient; used in eruptions.

Laugh Neagh, Down, said to cure running ulcers.

Madrid, a mineral water there, contains sulphate of soda very largely.

Mallow, Cork, is similar to Bristol; discovered 1689, by Dr. Rogers, of Cork, one of whose patients drank of the water accidentally. It discharges twenty gallons in the minute; is clear and limpid when drawn from the fountain, with a vapour arising from it. I have seen the most decided good effects from this water in consumptive habits, even in cases which were considered hopeless. It is used in the same manner as the Bristol water.

Maherabeg, Kerry, is a saline aperient water.

Malvern, Worcestershire, contains carbonic acid, lime, and magnesia, united with carbonic and muriatic acids; is tonic and diuretic. Is used in muco-purulent affections of the bladder, in hectic, in consumptive cases, in bilious and female diseases, in scrofulous and chronic eruptions.

Markshall, Essex, same as Islington.

Maudley, Lancashire, sulphurous and saline.

Matlock, Derbyshire, similar to Bristol; temperature 66°.

Micham, Fermanagh, a sulphurous water.

Miers, France, aperient and diuretic.

Millar's Spa, Lancashire, same as Tunbridge.

Miltown Mallay, Clare, a chalybeate water.

Moffat, Annandale, Scotland, contains sulphur, muriate of soda, and an earth. Is alterative, diuretic, and purgative: is used as a bath.

Mont d'Or, France, same as Aix-la-Chapelle.

Mont Pallas, Cavan, a chalybeate water.

Montmorency, Paris, a sulphurous spring.

Monfrin, Nimes, France, is aperient and diuretic.

Mosshouse, Lancashire, same as Islington.

Moreton, Shropshire, similar to Holt.

Motte, near Grenoble, is a warm sulphurous water.

Neville Holt, Lancashire, contains carbonated earth and Epsom salts.

Naphtha, Russia, a chalybeate.

New Cartmel, Lancashire, saline and aperient.

Naples, a sulphurous and chalybeate water, near St. Luke's church.

Newnham Regis, Warwickshire, similar to Scarborough.

Newtondale, Yorkshire, contains carbonate of lime and magnesia.

Newtown Stewart, Tyrone, is similar to Tunbridge.

Nezdenice, Germany, carbonate of iron and soda; diuretic and tonic.

Nobber, Meath, is similar to Hartfell.

Normanby, Yorkshire, is similar to Askeron water.

Nottington, Dorsetshire, sulphurous and saline; used in eruptions.
Oakfield, Cavan, a chalybeate.
O'Brien's Bridge, Clare, a strong sulphurous water.
Oersten, Denmark, little frequented.
Orston, Nottingham, contains carbonic acid in excess, iron, and sulphate and muriate of soda. It induces intoxication.
Oulton, Norfolk, same as Islington.
Owen Bruen, Cavan, same as Askeron.
Pancras, London, contains sulphate of magnesia and carbonated earth.
Peterhead, Aberdeen, a strong chalybeate.
Passi, near Paris, similar to Pyrmont and Cransac.
Perekop, Russia, a chalybeate.
Pettigae, Donegal, same as Askeron.
Phœnix Park, Dublin, a chalybeate and sulphurous water.
Pisa, Italy, a warm spring; also famed for baths.
Pithkeatly, Perthshire, Scotland, contains muriate of soda and an earth.
Plombieres, Lorraine, France, a saline and sulphurous warm spring; used in affections of bladder and asthma; and externally, in scrofula and cutaneous diseases.
Pontgibault, France. There are thirty-two springs here, whose temperatures vary from 82° to 124°: their effects are diuretic and laxative.
Pongues, Nivernois, France, contains calcareous earth, magnesia, carbonate of soda, muriate of soda, and silicious earth; diuretic and aperient.
Pyrmont, Westphalia, contains carbonate of iron, calcareous earth, magnesia, sulphate of magnesia, and muriate of soda. It is diuretic, diaphoretic, and aperient; and used in female complaints, in relaxed habits, in cutaneous and nervous diseases, urinary obstructions; and considered the best restorative in broken constitutions.
Prussia, Warmburn; the only mineral water there.
Queen Carmel, Somersetshire, contains sulphur, muriate of soda, and calcareous earth; is used in scrofulous and cutaneous affections.
Rykum, Iceland, contains pure soda.
Richmond, Surrey, same as Acton.
Rippon, Yorkshire, sulphurous and saline.
Riviera de Abajo, Spain, near Oviedo; said to be similar to Bath.
Road, Wiltshire, a sulphurous and chalybeate water; very strong.
St. Amands, Valenciennes, sulphurous; is used in eruptions, calculous cases, and piles.
St. Bartholomew's, Cork, same as Tilbury.
St. Bernard's, Edinburgh, is a strong sulphurous water, same as Harrogate; and is used in scorbutic and scrofulous diseases.
St. Erasmus' Well, Staffordshire, same as Barrowdale.
St. Windfrede's, Wiltshire, resembles Malvern.
Sarepta, Russia, a chalybeate.
Scarborough, Yorkshire, contains carbonate of lime, sulphates of magnesia and soda, and iron; is diuretic and purgative.

Scolliensis, Switzerland, contains iron, carbonate of soda, and carbonic acid.

Scool, Clare, is a chalybeate.

Sedlitz, Bohemia, contains sulphate of magnesia, and is strongly purgative; is used in stomach, nervous, hemorrhoidal, and œdematous complaints, and in anomalous cases succeeding cessation of the menses.

Seltzer, Germany, contains carbonic acid, carbonate of soda, calcareous earth, and magnesia; is used in gravel, hæmaturia, scurvy, scrofula, dyspepsia, heartburn, acidity, and in bilious and calculous disorders.

Sene, or Seude, Wiltshire, is similar to Islington.

Scydschutz, Germany, same as Sedlitz.

Shadwell, near London, contains sulphate of iron.

Shapmoor, Westmorland, is sulphurous and saline; same as Askeron.

Shettlewood, Derbyshire, is similar to Harrogate.

Shipton, Yorkshire, same as last named.

Skibbereen, Cork, a sulphurous water.

Somersham, Huntingdonshire, contains sulphate of iron; applied to old ulcers.

Spa, Germany, contains carbonate of soda, iron, carbonated earth, sulphate of magnesia, and muriate of soda; is used in suppression and retention of menses, hysterical and dropsical affections, and fluor albus.

Spain contains several waters of little note.

Stanger, Cumberland, contains sulphate of iron.

Stenefield, Lincolnshire, similar to Orston.

Streatham, Surrey, contains carbonated earth, sulphate and muriate of magnesia.

Suchaldza, Hungary, similar to Nezdenice.

Sweden mineral waters, said to contain muriate of potass.

Swansea, Glamorganshire, similar to Shadwell.

Swabia, Baden, is a warm sulphurous water; temperature 132°.

Sydenham, Kent, is similar to Epsom.

Tarleton, Lancashire, similar to Scarborough.

Tewksbury, Glocestershire, similar to Acton.

Thetford, Norfolk, contains carbonate of soda and iron, and carbonic acid.

Thorston, Nottingham, same as Orston.

Thursk, Yorkshire, same as Scarborough.

Tilbury, Essex, contains carbonate of soda; is diuretic and diaphoretic.

Tibshelf, Derbyshire, is similar to Spa.

Toberbony, Dublin, is an alkaline water.

Toustein, Germany, is similar to Seltzer.

Toplitz, Bohemia, a saline water; famed also for baths.

Tralee, Kerry, is similar to Castleconnel.

Tunbridge, Kent, contains iron, muriate of soda, and calcareous earth; used in all diseases in which chalybeates are serviceable.

Turkey, waters of, very little known.

Vals, Dauphiny, contains carbonate of soda; is diuretic and diaphoretic.

Vezoul, Upper Saone, is aperient, refrigerant, and diuretic.
Vechy, near Moulins, contains carbonate of soda and sulphate of iron; is used in jaundice, affections of the kidney and bladder, and in sterility.
Viterbo, in Italy, said to contain sulphurous acid.
Wardrew, Northumberland, is similar to Harrogate.
Wallenpow, Northamptonshire, same as Islington.
West Ashton, Wiltshire, similar to last named.
Westwood, Derbyshire, same as Shadwell.
Wexford Spa, is said to be like Islington.
Wisbaden, Germany, a carbonated water; is famed for baths.
Whiteacre, Lancashire, contains carbonate of iron and lime.
Wildungan, Germany, said to be similar to Bath.
Wildbad, Germany, famed for warm baths.
Witham, Essex, contains carbonate of iron and muriate of soda.
Wirksworth, Derbyshire, sulphurous, saline, and chalybeate.
Wurtemburg, Germany, famed for baths.
Zahorovice, Germany, similar to Nezdenice.

